# Using the WHO safe childbirth checklist to improve essential care delivery as part of the district-wide maternal and newborn health quality improvement initiative, a time series study

**DOI:** 10.1186/s12913-021-06781-x

**Published:** 2021-08-16

**Authors:** Befikadu Bitewulign, Dereje Abdissa, Zewdie Mulissa, Abiyou Kiflie, Mehiret Abate, Abera Biadgo, Haregeweyni Alemu, Meseret Zelalem, Munir Kassa, Gareth Parry, Hema Magge

**Affiliations:** 1 Institute for Healthcare Improvement, Addis Ababa, Ethiopia; 2 Oromia Regional Health Bureau, Addis Ababa, Ethiopia; 3 Minstry of Health-Ethiopia, Addis Ababa, Ethiopia; 4 Department of Plastic and Oral Surgery, Boston Children’s Hospital, Boston, MA USA; 5 Bill and Melinda Gates Foundation, Seattle, USA; 6 Division of Global Health Equity, Brigham and Women’s Hospital, Boston, MA USA; 7 Division of General Pediatrics, Boston Children’s Hospital, Boston, MA USA

**Keywords:** Quality of care, Clinical bundle, World Health Organization safe childbirth checklist, Ethiopia, Maternal and newborn health

## Abstract

**Background:**

Care bundles are a set of three to five evidence-informed practices which, when performed collectively and reliably, may improve health system performance and patient care. To date, many studies conducted to improve the quality of essential birth care practices (EBPs) have focused primarily on provider- level and have fallen short of the predicted impact on care quality, indicating that a systems approach is needed to improve the delivery of reliable quality care.

This study evaluates the effect of integrating the use of the World Health Organization Safe Childbirth Checklist (WHO-SCC) into a district-wide system improvement collaborative program designed to improve and sustain the delivery of EBPs as measured by “clinical bundle” adherence over-time.

**Methods:**

The WHO-SCC was introduced in the context of a district-wide Maternal and Newborn Health (MNH) collaborative quality of care improvement program in four agrarian Ethiopia regions. Three “clinical bundles” were created from the WHO-SCC: On Admission, Before Pushing, and Soon After Birth bundles. The outcome of each bundle was measured using all- or- none adherence. Adherence was assessed monthly by reviewing charts of live births.

A time-series analysis was employed to assess the effectiveness of system-level interventions on clinical bundle adherence. STATA version 13.1 was used to analyze the trend of each bundle adherence overtime.

Autocorrelation was checked to assess if the assumption of independence in observations collected overtime was valid. Prais-Winsten was used to minimize the effect of autocorrelation.

**Findings:**

Quality improvement interventions targeting the three clinical bundles resulted in improved adherence over time across the four MNH collaborative. In Tankua Abergele collaborative (Tigray Region), the overall mean adherence to “On Admission” bundle was 86% with β = 1.39 (95% CI; 0.47–2.32; *P* <  0.005) on average monthly.

Similarly, the overall mean adherence to the “Before Pushing” bundle in Dugna Fango collaborative; Southern Nations, Nationalities and People’s (SNNP) region was 80% with β = 2.3 (95% CI; 0.89–3.74; *P* <  0.005) on average monthly.

**Conclusion:**

Using WHO-SCC paired with a system-wide quality improvement approach improved and sustained quality of EBPs delivery. Further studies should be conducted to evaluate the impact on patient-level outcomes.

## Background

Poor quality care during institutional births, particularly in low and middle-income countries (LMIC), has been recognized as a major contributing factor to childbirth-related harms as care providers may fail to execute essential birth practices (EBPs) in real time [1].

The ‘know-do’ gap – the difference between a provider’s knowledge and behavior – has often been cited as a phenomenon in care delivery, which many believe may relate to the failure to remember critical steps during clinical care [2–5].

Checklists have been used as a tool to improve healthcare worker practices to deliver high quality essential care during institutional births [6, 7]. However, evidence shows that when implemented alone they may not result in change or improvement in quality of care [4]. Furthermore, studies show that the provision of clinical guide (tools) trainings to frontline healthcare providers alone will not be sufficient to improve adherence of core clinical care practices at required level [8].

In complex health system, determining the best way to translate novel checklists to improve adherence to evidence based practices by the end users may require system redesign at multiple interconnected levels, including behavioral change interventions [9–11].

System level approach led by government engaging all levels of the health system is recommended to effectively implement effective interventions at scale in LMICs [10, 12, 13].

To help skilled birth attendants (end-users) remember EBPs in real time and adhere to it, the World Health Organization Safe Childbirth Checklist (WHO-SCC) was developed by WHO and partners [14]. The Checklist is an organized list of evidence -based essential birth practices which guides the end-uses to pause and check at four critical points during childbirth: On Admission, Before Pushing (or before Caesarian), Soon After Birth (within 1 h), and Before Discharge. The checklist was designed to address the major causes of maternal and neonatal deaths [14, 15].

Based on promising preliminary results to improve EBP delivery, “The Better Birth Trial”- was designed to measure the impact of the WHO-SCC. There was no significant effect found on maternal or perinatal mortality or maternal morbidity despite having positive effects on EBPs during the intervention. Furthermore, adherence to EBPs was not sustained beyond the intervention period when the coaches were absent [16, 17]. The authors suggest that provider-level interventions may not fully translate into improved patient outcomes if not incorporated into a broader system-level improvement across facilities and referral systems.

A study implemented in Rwanda using WHO-SCC found an overall improvement in the EBPs compliance rate. Significant improvements were seen in 11 out of 29 EBPs. The reasons for low compliance to other EBPs were not identified even though clinical care providers received training on the use of WHO-SCC prior to implementation [18]. These results indicate that systems improvement efforts may be required to close remaining gaps and achieve high enough reliability of adherence to achieve change in patient outcomes.

Clinical bundles have been developed and used in improvement science efforts as an approach to achieve system level change. A clinical bundle is defined as a small set of evidence-based interventions for a defined clinical domain that when implemented together at high reliability, will result in significantly better outcomes than when implemented individually [19].

Bundles are thought to promote awareness that an entire care team must work together in a system designed for reliability. Bundles also promote the use of improvement methods to redesign care processes [19].

Using standard quality improvement (QI) methods, bundles have been found to drive performance to new levels with the theory that in order to achieve high levels of reliable bundle implementation it will require fundamental system change which lead to better and sustained results [20–24]. For instance, if each of five bundle elements are delivered at 90% reliability, then the bundle is reliably delivered at 59%, as bundle reliability is the product of each element’s reliability (90% × 90% × 90% × 90% × 90%) [25]. Studies indicate that all-or-none bundle measurement can help achieve new levels of performance and improved patient outcomes [20, 26].

The Institute for Healthcare Improvement (IHI) in partnership with the Ethiopian Ministry of Health (MOH) integrated the Ethiopian-adapted WHO-SCC checklist into a broader district-wide MNH quality of care (QoC) improvement effort, with the ultimate aim of improving QoC and reducing maternal and newborn mortality.

This study evaluates the effect of integrating the MOH-adapted WHO-SCC into a broader district-wide system improvement collaborative program. The program was measured by clinical bundle adherence over time in four collaborative of Ethiopia’s major regions: Oromia, Amhara, Southern Nations Nationalities People (SNNP), and Tigray.

## Methods

### Program description

**Fig. 1:**
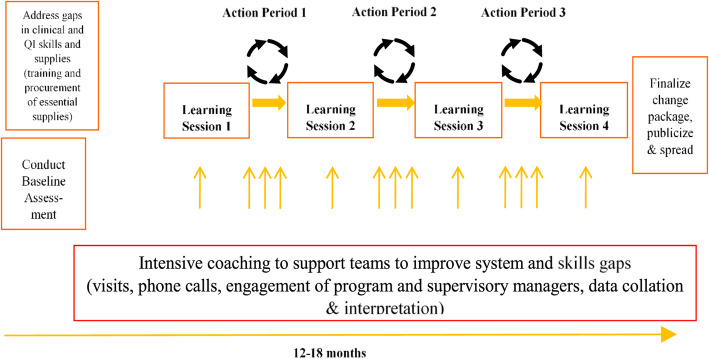
IHI's Improvement Collaborative Design

The WHO-SCC was introduced in the context of a large-scale QI program being tested within the Ethiopian public health system. This intervention used a district-wide improvement collaborative designed to improve the quality of maternal and newborn health (MNH) care. The collaborative design (Fig. 1) was based on IHI’s Breakthrough Series collaborative model. The goal of the collaborative is to convene a group of facilities around accelerating improvement in a common priority area using improvement methods and an established learning network [27].

The improvement collaborative were aligned to the administrative structure of the district, and had the following basic elements: selection of priority area and target indicators, QI training for QI teams, baseline data collection, and action plans to address key gaps in essential commodities and clinical skills.

IHI was requested by the Ethiopian MOH to include the adapted version of WHO-SCC introduction as part of the Maternal and Newborn Health (MNH) QI effort. The adaptation was made to address major causes of maternal and neonatal morbidity and mortality in Ethiopian context. For example, does the mother/baby need to start antiretroviral drug is included as an element across the pause points.

This checklist was introduced to collaborative health care facilities during the initiation of the program as a reminder for clinical care providers to practice EBPs in real-time. The facility level end users initially used the “READ-DO” approach; read the checklist first and accomplished the EBP’s. Later after earning ample experience in utilization of the checklist, they used the “Do-Confirm” approach; completed the task then read the item on the Checklist to confirm that the care is practiced and ticked. The facility QoC improvement teams designed a number of improvement projects to maximize real time utilization of the checklist. The Coaching teams included support for WHO-SCC use with patients and QI support for projects aimed to improve system performance measured by clinical bundles.

Adequate orientation for the proper use of the WHO-SCC was given to facility QI teams as part of the QI initiative and implemented in line with similar studies in LMIC [18, 28, 29].

The program team collaborated with professional associations to support clinical trainings such as Helping Babies Survive (HBS) and Basic Emergency Obstetric Newborn Care (BEmONC) as needed. Subsequently, QI teams from health centers and hospitals within each district convened in a series of “learning sessions”. This is intermittent face-to-face meetings with facility QI teams and leaders to share their progress, challenges, receive targeted QI support and share critical learnings from the testing process. Between learning sessions, facility teams implemented their QI projects using the Model for Improvement (MFI) as a framework for developing, testing, and implementing changes in a system to improve process reliability and outcomes of interest [30].

Teams tested newly developed change ideas and received on-site integrated clinical/QI coaching support from joint IHI-district leadership coaches. The collaborative was organized in four sessions during a 12–15-month period in the selected collaborative.

### Setting and site selection

In Ethiopia, maternal and neonatal mortality remain unacceptably high at 412 deaths per 100,000 live births and 29 deaths per 1000 live births, respectively; neonatal mortality accounts for about 43% of all under-5 deaths in Ethiopia and has had the slowest decrease over the past 16 years in comparison with the rest of child mortality [31]. These gaps are due to both utilization and quality issue, e.g. only 26% of deliveries are attended by a skilled birth attendants; and less than half of mothers receive any clinical check-up during and after delivery [10, 31]. To address these needs, the Ethiopian Ministry of Health (MoH) advocated for quality and equity as a core pillar in its 2015 Health Sector Transformation Plan (HSTP) [32], to achieve improved health outcomes at scale. In line with this, we co-designed the Ethiopia Healthcare Quality Initiative to accelerate health system across the country using a phased design for scale. The first phase of the program was implemented in one district improvement collaborative at Tankua Abergele, Dugna Fango, Lemmu Bilbilo and Fogera collaborative located in the regions of Tigray, SNNP, Oromia, and Amhara respectively (four of Ethiopia’s most populous regions).

All facilities in each district were included to ensure a district-wide approach, which consisted of three primary hospitals and twenty-seven health centers across the four district improvement collaborative. Collaborative were selected by regional leadership based on need for improvement, lack of other MNH partner-supported initiatives and the local leadership’s desire for the approach. Leaders from the collaborative also demonstrated commitment to generate honest data for improvement.

### Outcome measures

In consultation with MOH-MNCH Directorate, we designed three clinical bundles selected from the WHO-SCC (Table 1). The three clinical bundles were measured using all-or-none bundle adherence (adherence = yes if all bundle elements achieved) to include among the collaborative target indicators.

**Table 1:** Elements of the clinical bundle extracted from MOH adapted Safe Childbirth Checklist

Clinical Bundles	Safe Childbirth Checklist Bundle Element
**On Admission Bundle**	Danger sign assessment
	Partograph initiated when cervical dilation at least 4 cm
	Availability of soap, water, alcohol hand rub and gloves
	Birth companion encouraged to be present during labor and at birth
	Mothers privacy maintained during labor and delivery
**Before Pushing Bundle**	Availability of gloves, soap/savlon and clean water
	Preparation of 10 IU IV/IM Oxytocin in syringe
	Availability of two clean, dry, warm towels and suction device
	Availability of bag and mask (size 0 and 1)
	Helper/Assistant identified and informed for resuscitation
**Soon After Birth Bundle (within 1 h)**	Newborn assessment
	Immediate skin to skin and initiate breastfeeding within the 1st hour
	Baby weighed and recorded
	Administer Vitamin K1
	Administer tetracycline eye ointment

The selection of the clinical bundle elements to create the “all or none bundles” was made using a defined criteria’s set by pool of high level clinical and quality improvement experts from Ethiopian Ministry of Health and Institute for Healthcare Improvements. The co-developed clinical bundles are based on content derived from the standard Ethiopian protocols and WHO Safe Childbirth Checklist. Some of the criteria’s used was availability of data on individual patient folder care documentation for the purpose of triangulating against the bundle element ticked on the WHO Safe Childbirth Checklist for measurement. Above all, the ability of the selected bundle to improve the general preventive care and to impact the birth outcome was considered.

The outcome measures for this study are all-or-none adherence to On Admission, Before Pushing and Soon After Birth bundles.

### Data collection

The data sources included audits of WHO-SCC and medical records. In health facilities where the number of monthly deliveries were greater than 30, a systematic random sampling method was used to retrieve 30 charts to calculate all-or-none bundle adherence using an excel template design as part of the program monitoring tool. In health centers where the number of facility births was less than 30, the total number of monthly deliveries was selected to calculate bundle adherence.

To determine adherence to a set of checklist practices, triangulation of documented bundle element from individual patient/client folder with the Safe Childbirth checklist during the chart review process was made. If all elements have been documented on patient folder and ticked on the WHO-SCC check box, the bundle is counted as complete for that patient and is scored as “1”. If any of the elements are absent either on patient documentation or not ticked on the WHO-SCC, the bundle is incomplete (no “partial credit” is given) thus it is scored “0”.

At the end of each month, senior project officers (SPOs), District level Quality Improvement coaches and respective facility leaders who were trained well on both the adapted WHO-SCC and the bundle data collection tool were involved in the review process. In addition, real time observation of the checklist was made by Senior Project Officers from IHI and respective system leaders during periodic joint coaching visits.

On a monthly basis, the data from respective collaborative health facilities were aggregated to create collaborative wide all- or-none bundle adherence—a dependent variable of our study.

The study period in Oromia, Tigray and SNNP was from November 2016 to December 2018. Unlike other regions, the start date of collaborative in the Amhara region was delayed by 7 months due to political instability in the region. As the result, the study period was June 2017 to December 2018.

No baseline data were collected before the start of the intervention (study period) because the WHO-SCC was introduced for the first time as part of the quality improvement program.

### Analysis

The trend of adherence to each clinical bundle over time was analyzed from the collaborative start date to the end of the project. Sustainability was assessed using a follow-up period of 12 months for all collaborative except Fogera (Amhara region).

For each clinical bundle, a time series analysis using STATA version 13.1 was used to assess the effect of system-level interventions on all-or-none bundle adherence over time for the four collaborative.

Durbin Watson statics—a test for autocorrelation in the residuals from a statistical regression analysis was used to check if the assumption of independence in observations collected over time was valid. To fit the purpose, monthly collaborative- wide clinical bundle adherence mean was calculated and equally spaced for respective district. Furthermore, Prais-Winsten — a procedure meant to take care of the serial correlation of type Auto -regression (AR (1)) in a linear model — was used to minimize the effect of autocorrelation.

## Results

Table 2 describes the characteristics of study collaborative and the interventions. Facility-level QI teams received an average of about 20 coaching visits throughout the intervention period with some variability. This achieved the program’s target which was to hold at least one joint coaching visit per month per district.

**Table 2:** Characteristics of MNH quality improvement collaborative prototype collaborative with interventions. November 2016–December 2018, Ethiopia

Characteristics	Tigray Region	Amhara Region	Oromia Region	SNNPR Region	Mean	Standard Deviation
District	Tankua Abargele	Fogera	Lemmu Bilbilo	Dugna Fango		
Total number health centers	5	10	7	5		
Total number primary hospitals	1	0	1	1		
Geographical characteristics	Agrarian	Agrarian	Agrarian	Agrarian		
Total population (beginning of project)	115,841	296,844	213,032	122,316		
Total number of learning sessions conducted	4	4	4	4		
Total Number of healthcare providers per collaborative	89	126	112	88	103	18.5
Total Number of healthcare providers for childbirth per collaborative	21	28	31	14	23	7.5
Total Number of healthcare providers for childbirth per collaborative trained on QILM*	7(33%)	10(36%)	9(29%)	7(50%)	8	1.5
Average number of coaching/mentoring visits received per QI team/facility/month	2.1	1.3	1.2	1.8	1.6	0.42
Number of health care providers trained on BEmONC* per collaborative district	15	11	11	6	11	3.6
Number of health care providers trained on HBS* per collaborative district	16	30	24	15	21	7
Number of health care providers trained on NICU* per hospital	5	4	5	5	4.7	0.5
Number of system leaders trained on QILM per collaborative	8	12	10	8	9.5	1.9
Total number of Skilled birth attendance (Sep-2016-June 2018) per collaborative	4453	7410	7468	7166	6624	1453
Total number of Skilled birth attendance (Sep-2016-June 2018) per Hospital	1574	0*	2734	2017	1581	1157

*BEmONC Basic Emergency Obstetrics and Newborn Care *HBS Helping Baby Survive *NICU Neonatal Intensive Care Unit *QILM Quality Improvement and Leadership Management *0- There is no primary Hospital in Fogera Collaborative

**Fig. 2:**
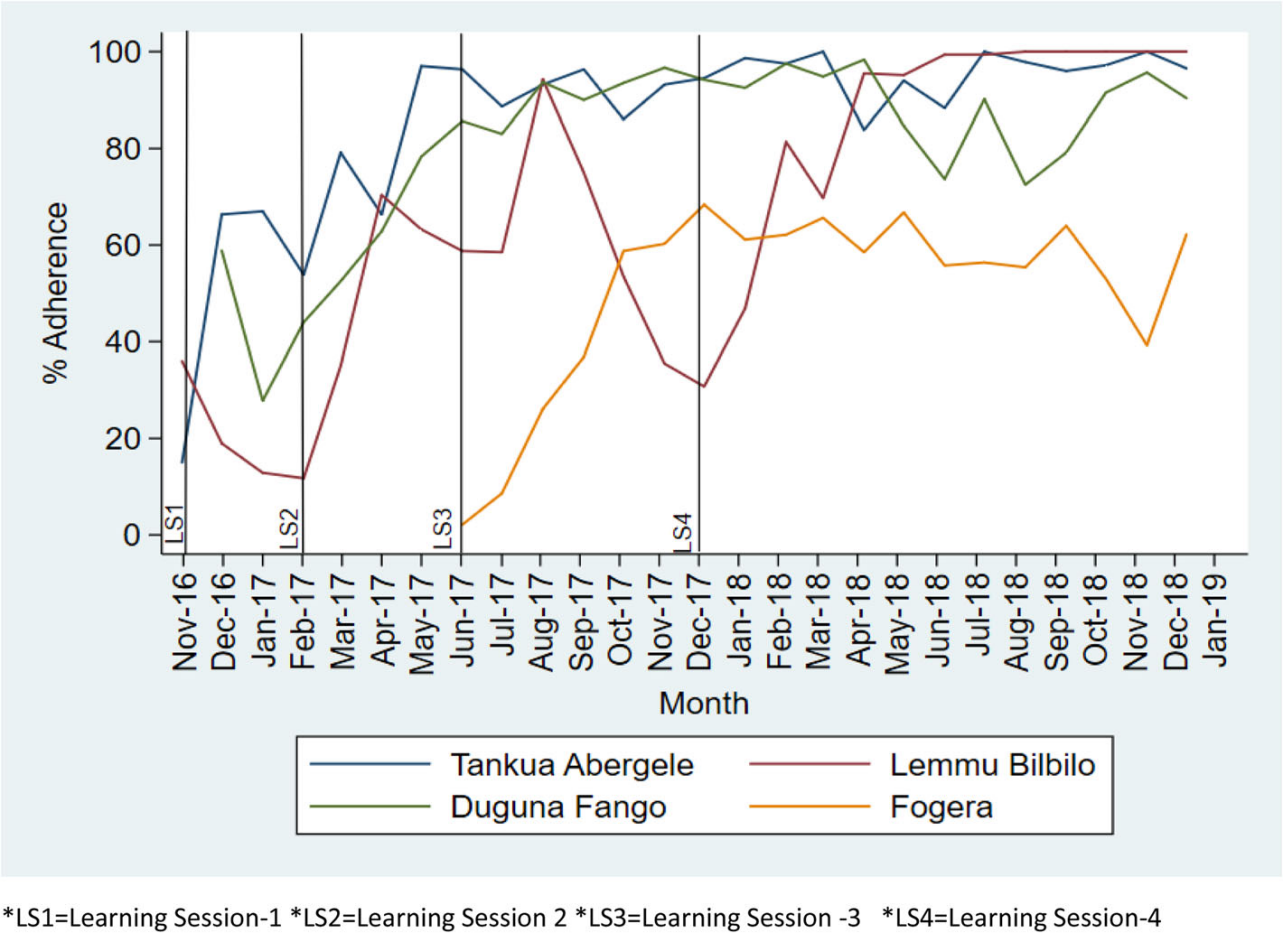
Trend of all or none bundle adherence to On Admission bundle across the four collaborative. *LS1 = Learning Session 1 *LS2 = Learning Session 2 *LS3 = Learning Session 3 *LS4 = Learning Session 4

**Fig. 3:**
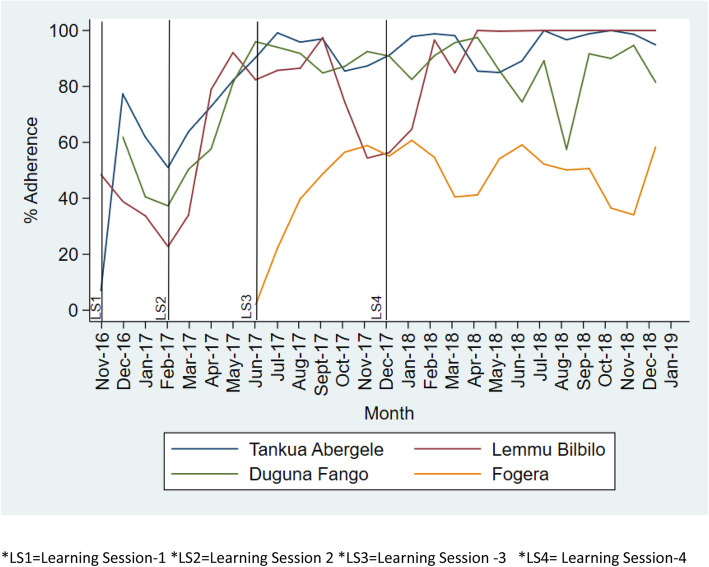
Trend of all or none bundle adherence to Before Pushing bundle across the four collaborative. *LS1 = Learning Session 1 *LS2 = Learning Session 2 *LS3 = Learning Session 3 *LS4 = Learning Session 4

**Fig. 4:**
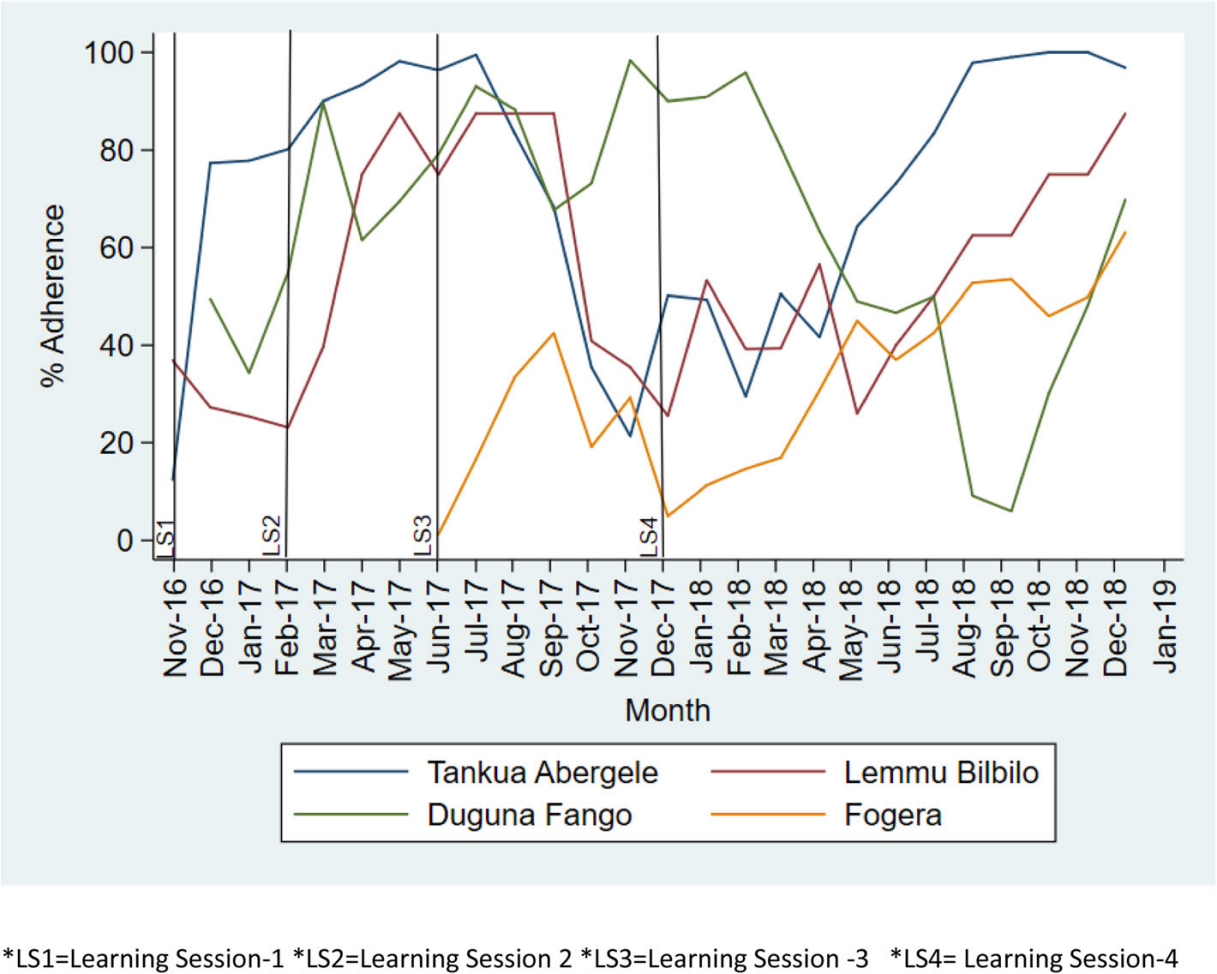
Trend of all or none bundle adherence to Soon After Birth bundle across the four collaborative. *LS1 = Learning Session 1 *LS2 = Learning Session 2 *LS3 = Learning Session 3 *LS4 = Learning Session 4

All-or-none bundle adherence to On Admission, Before Pushing, and Soon After Birth bundles in all collaborative have shown a positive monthly adherence increment (Figure 2, 3, 4). For instance, in Tigray region, Tankua Abargele collaborative, the overall mean adherence to on Admission bundle was 86% with β = 1.4 (95% CI; 0.47 4–2.3) on average monthly (Table 3); which implies for every quality improvement intervention (QI training, learning sessions, coaching visit etc.) made across months, adherence to the on Admission bundle was increased by 1.4.

**Table 3:** Prais-Winsten AR (1) regression coefficients result of On Admission, Before Pushing and Soon Afterbirth Bundles across district. November 2016–December 2018, Ethiopia

Collaborative District	On admission Bundle				Before Pushing Bundle				Soon After Birth Bundle			
	Constant		Slope		Constant		Slope		Constant		Slope	
	Coefficient	P	Coefficient (95% CI)	P	Coefficient (95% CI)	P	Coefficient (95% CI)	P	Coefficient (95% CI)	P	Coefficient (95% CI)	P
	(95% CI)											
TankuaAbergele	69.7 (55.4,83.9)	< 0.001	1.4 (0.47, 2.32)	0.005	69.6 (51.5,81.8	< 0.001	1.5 (0.47, 2.32)	< 0.005	55.65 (5.6,97.7)	0.03	1.4 (−1.41, 4.3)	0.308
Dugna Fango	46.1 (26.9, 65.3)	< 0.001	2.3 (1.1, 3.5)	< 0.005	44.24 (21.8, 66.6)	< 0.001	2.3 (0.89, 3.7)	< 0.005	57.3 (9.53, 105)	0.021	0.3 (−2.7,3.3)	0.841
Lemmu Bilbilo	26.2 (−13.63, 66.04)	0.18	2.9 (0.86, 4.8)	0.02	26.27 (−4.31,57.86)	0.088	2.8 (0 .86, 4.8)	0.008	58.7 (29.2,88.2)	< 0.001	0.7 (−1.2,2.54)	0.464
Fogera	50.1 (22.85, 77.4)	22.85	0.12 (1.6, 1.9)	1.4	53.2 (22.85, 77.4)	< 0.001	0.31 (−.83,1.4)	0.579	58.1 (47.5, 68.8)	< 0.001	0.1 (−.45,0.74)	1.4

Similarly, the overall mean adherence to Before Pushing in SNNP region, Dugna Fango collaborative was 80% with β = 2.3 (95% CI; 0.89–3.7) on average monthly (Table 3). This implies that for every quality improvement intervention (QI training, learning sessions, coaching visit etc.) made across months, adherence to the Before Pushing bundle adherence was increased by 1.4.

The overall mean adherence to the Soon After Birth bundle in Amhara region (Fogera collaborative) and Oromia region (Lemmu Bilbilo collaborative) was 32% with β = 0.15 (95% CI; − 0.45 - 0.74) and 20% with β = 0.7 (95% CI; − 1.2 - 2.5) on average respectively/month (Table 3). This implies for every quality improvement intervention (QI training, learning sessions, coaching visit etc.) made across months, adherence to the Soon Afterbirth bundle was increased by 0.15 and 0.7 for Fogera collaborative and Lemmu Bilbilo collaborative respectively.

In addition, adherence to the clinical bundles was sustained in all collaborative beyond the intervention period (December 2017 to December 2018) (Figs. 2, 3, 4).

## Discussion

To the best of our knowledge, using the all-or-none bundle approach to measure adherence to evidence-based EBPs extracted from the WHO-SCC is the first of its kind. System- level interventions through the integration of the WHO-SCC into the district-wide MNH QI collaborative program has led to a marked increase in delivery of EBPs over time.

This has been made evident by improved adherence to On Admission, Before Pushing and Soon After Birth bundles both during the intervention period and for 12 months after the intervention period. The sustained improvement could indicate integration of changes into the routine system and ownership of the quality improvement approach.

Our study has several strengths. A standardized WHO-SCC was used to facilitate quality care in the context of a guided approach with clinical mentorship, measurement introduction, data collection, monitoring and response in a variety of health facilities across a large geographic area of rural Ethiopia. Monthly data was collected allowing for a time-series analytic approach, which can be a rigorous way of assessing change using routine programmatic data.

The On Admission and Before Pushing bundles were highly reliable in all study collaborative. However, a marked drop in adherence to the Soon after birth bundle was observed from October 2017–December 2017 at Lemmu Bilbilo, which we believe was due to political instability in the district which caused disruption in the supply chain of Vitamin K and tetracycline eye ointment from the regional capital to the district.

One possible explanation for the higher levels of reliability of the On Admission and Before Pushing bundles could be due to the fact that elements in both bundles like liquid soap, gloves and others are available in health facilities store and accessible from local markets. During the baseline assessment, oxytocin is over stocked in most facilities whereas stock of Vitamin K, TTC eye ointment was sub optimal. Due to low demand from health facilities’ the national level stock of Vitamin K is low compared to other essential drugs which might be related to low habit of Vitamin K demand request by health facilities. In addition, the private drug venders have low stock of Vitamin K due to low demand in the market.

All-or-none adherence to the Soon After Birth bundle across all regions took a considerable time to achieve a higher level of reliability. This is primarily due to the shortage of Vitamin K and the lengthy procurement process to purchase Vitamin K from private drug vendors. In response, facility QI teams have shifted focus of QI efforts onto supply chain measurement as a result significant improvement was observed after the period of low compliance (October–December 2017) across all collaborative.

Following the introduction of the WHO-SCC in the MNH QoC improvement collaborative facilities, the health care workers were able to identify and document newborns with complications and initiate higher level care in the effort to reduce mortality, a common recommendation of many studies [33–35]. This, in turn, led to the establishment of level II neonatal intensive care units (NICU) and implementation of feasible evidence-based interventions such as kangaroo mother care at 3 primary Hospitals of the three collaborative.

While we used bundle adherence to reliably improve EBPs extracted from the WHO-SCC, adherence to individual EBPs also significantly improved during the intervention period and was consistent with other studies [6, 18, 36, 37]. However, unlike other studies [16, 17], adherence to EBPs was sustained in our program beyond the intervention period.

This could be attributed to the engagement of local leadership from the baseline assessment to the fourth learning session, enablement of local ownership via joint coaching visits, ensuring local relevance and acceptability by running multiple Plan-Do-Study-Act cycles (PDSAs) before initial implementation of the WHO-SCC, and creating the intrinsic motivation of the end-users for successful adaptation of the WHO-SCC. This comprehensive behavior change strategy facilitated by our program has led to habits of continuous QI across the system as evidenced by incremental and sustained adherence to the three clinical bundles over time.

Our study has a number of limitations. Due to feasibility constraints and the nature of the quality improvement methodology in which QI teams ideally own the data collection and analysis themselves, we were limited to the use of routinely available data.

Comparison facilities were not included in this study due to feasibility. Finally, due to the small volume of facilities, measuring impact on neonatal mortality was not feasible, and is the subject of a larger program evaluation.

## Conclusion

Embedding the use of the WHO-SCC with rigorous measures and system improvement methods to address system gaps beyond the individual provider-patient interaction could be a promising approach to improving the delivery of essential MNH interventions. Further study is underway to evaluate impact on patient-level outcomes.

## Data Availability

The datasets used during the current study are available from the Institute for Healthcare. Improvement on reasonable request.

## Abbreviations

AC: Autocorrelation
BEmONC: Basic emergency obstetric and newborn care
EBP: Essential birth practice
MOH: Ministry of Health
HBS: Helping Babies Survive
IHI: Institute for Healthcare Improvement
LMIC: Low and middle income countries
MFI: Model for Improvement
MNH: Maternal and newborn health
QILM: Quality improvement, leadership and management
QI: Quality improvement
QoC: Quality of care
SNNP: Southern Nations Nationalities and People
WHO-SCC: World Health Organization Safe Childbirth Checklist
